# Antimicrobial resistance phenotypes, disinfectant susceptibility, and resistance-associated gene detection in carbapenem-resistant Pseudomonas aeruginosa from two tertiary hospitals in Urumqi, China

**DOI:** 10.3389/fcimb.2026.1938496

**Published:** 2026-09-21

**Authors:** Ping Tian, Ke Niu, Jie Li, Tingting Guo, Li Liu, Xiao Wang, Adila Maimaiti

**Affiliations:** 1 Department of Medical Administration, The Fifth Affiliated Hospital of Xinjiang Medical University, Urumqi, Xinjiang, China; 2 Xinjiang Regional Population Health Research Center, Urumqi, Xinjiang, China; 3 College of Nursing, Xinjiang Medical University, Urumqi, Xinjiang, China

**Keywords:** antimicrobial resistance, carbapenem-resistant Pseudomonas aeruginosa, disinfectant susceptibility, extensively drug-resistant, OprD, qacEΔ1-su1

## Abstract

**Objective:**

To characterise antimicrobial resistance phenotypes, *in vitro* susceptibility to four commonly used disinfectants, and the distribution of resistance-associated genes among clinical carbapenem-resistant Pseudomonas aeruginosa (CRPA) isolates from two tertiary hospitals in Urumqi, China, in order to provide local surveillance evidence to inform antimicrobial stewardship and infection prevention and control.

**Methods:**

Eighty nonduplicate CRPA isolates were consecutively recovered from clinical specimens at two tertiary hospitals in Urumqi between January 2025 and March 2026. Antimicrobial susceptibility was determined using the VITEK 2 system, with disk diffusion performed when confirmatory testing was required, and interpreted according to CLSI M100-S31 (2021). Carbapenemase production was assessed by the modified carbapenem inactivation method (mCIM). A broth microdilution assay adapted from antimicrobial susceptibility testing, and not a standardised biocide efficacy test, was used to compare minimum inhibitory concentrations (MICs) and minimum bactericidal concentrations (MBCs) of benzalkonium bromide, ethanol, povidone-iodine, and a chlorine-containing disinfectant between isolate groups. Polymerase chain reaction (PCR) was performed to detect carbapenem resistance-associated genes *(blaKPC, blaNDM, oprD, blaOXA-10, blaOXA-48, blaVIM, blaIMP*) and disinfectant resistance genes (*aceI, emrE, merA, qacEΔ1-sul1, tehA, oqxA*). Outcomes were organised into one primary and three exploratory families, with Holm and Benjamini–Hochberg correction applied within families respectively.

**Results:**

Of the 80 CRPA isolates, 24 (30.00%) were extensively drug-resistant (XDR) and 56 (70.00%) were non-XDR. Resistance rates to imipenem and meropenem were 83.70% and 68.70% respectively, and no isolate was susceptible to either agent. Overall resistance rates to aztreonam, piperacillin/tazobactam, levofloxacin, ceftazidime, cefepime, amikacin, and tobramycin were 37.50%, 30.00%, 26.30%, 25.00%, 21.30%, 12.50%, and 6.30% respectively. The expected oprD amplicon failed to amplify in 55 isolates (68.75%). mCIM was positive in 23 isolates (28.75%), with 95.0% agreement with PCR-detected carbapenemase gene carriage (Cohen’s κ 0.871, 95% CI 0.748–0.994). Detection rates for *blaKPC, blaNDM, blaVIM, and blaIMP* were 10.00%, 6.25%, 5.00%, and 2.50% respectively; neither *blaOXA-10* nor *blaOXA-48* was detected. *qacEΔ1-sul1, merA, tehA, and oqxA* were detected in 36.25%, 16.25%, 7.50%, and 2.50% of isolates respectively, whereas aceI and *emrE* were not detected. For ethanol the MIC50 and MIC90 were 75% (v/v) in both groups; for the chlorine-containing disinfectant and povidone-iodine the MIC50/MIC90 were 512/1024 mg/L in both groups, and neither MICs nor MBCs differed between groups. Benzalkonium bromide MICs ranged from 32 to 256 mg/L in XDR and 16 to 256 mg/L in non-XDR isolates, and MBCs from 64 to 512 and 64 to 256 mg/L respectively; the MIC difference was the primary outcome and remained significant after Holm correction (adjusted P < 0.001), whereas the MBC difference did not survive correction when considered alongside the other disinfectant endpoints (Benjamini–Hochberg q=0.144). qacEΔ1-sul1 was more frequent among XDR isolates in unadjusted analysis (58.3% versus 26.8%; OR 3.83, 95% CI 1.40–10.45; unadjusted P = 0.011) but did not retain significance after correction within the eight-test gene family (q = 0.088). Resistance to aztreonam and meropenem was nominally more frequent among *qacEΔ1-sul1*-positive isolates (55.20% versus 27.50% and 86.2% versus 58.80%) but neither association survived correction (q=0.072 for both; Holm-adjusted P = 0.111 and 0.100). Direct comparison of benzalkonium bromide endpoints by qacEΔ1-sul1 status showed no difference (median MIC 64 mg/L in both groups, P = 0.097; median MBC 128 mg/L in both groups, P = 0.697).

**Conclusions:**

CRPA isolates from these two hospitals showed uniform nonsusceptibility to carbapenems, with failure to amplify the expected oprD target the most frequent molecular observation. XDR isolates showed higher benzalkonium bromide MICs *in vitro* compared with non-XDR isolates. qacEΔ1-sul1 was the predominant disinfectant resistance gene detected, but its association with the XDR phenotype did not survive correction for multiple comparisons, and carriage was not accompanied by any measurable difference in benzalkonium bromide susceptibility. These findings provide baseline epidemiological data for Urumqi, though whole-genome sequencing, molecular typing, expression analysis, and standardised biocide efficacy testing would be required to establish mechanistic determinants.

## 1 Introduction

Antimicrobial resistance is a major threat to global public health and patient safety, and its associated clinical and economic burden continues to increase (Yano et al., 2024). Carbapenems have long been used to treat infections caused by multidrug-resistant Gram-negative bacteria because of their broad spectrum and potent bactericidal activity; however, widespread use has intensified selective pressure for resistance (Xu et al., 2025). Carbapenem-resistant Pseudomonas aeruginosa (CRPA) is a prominent cause of difficult-to-treat healthcare-associated infections and has been associated with prolonged hospitalization, greater morbidity, and an increased risk of death (Saha et al., 2022). Carbapenem resistance in P. aeruginosa is multifactorial and may involve carbapenemase production, loss or functional alteration of the outer-membrane porin OprD, and overexpression of several efflux systems. These mechanisms can act independently or in combination and may contribute to extensively drug-resistant (XDR) phenotypes (Bocharova et al., 2019).

Prolonged, inappropriate, or subeffective exposure to disinfectants in healthcare environments may also reduce bacterial susceptibility to some biocides. Certain efflux systems recognize multiple antimicrobial compounds, including quaternary ammonium compounds. In addition, antimicrobial and disinfectant resistance genes located on the same mobile genetic elements may be maintained under shared selective pressures, further complicating infection-control efforts (Ni et al., 2022).

Most systematic studies of CRPA in China have been conducted in the more densely resourced central and eastern regions, whereas data from northwestern China remain comparatively limited (Bai et al., 2024). Available evidence indicates that oprD alterations and acquired carbapenemase genes are important determinants of CRPA, while qacEΔ1-sul1 is frequently associated with class 1 integrons and may co-occur with multidrug resistance (Zhao et al., 2024). Some studies have further reported elevated disinfectant MICs and MBCs among multidrug- or extensively drug-resistant Gram-negative isolates (Menofy et al., 2025). These observations suggest that antimicrobial resistance and reduced disinfectant susceptibility may be linked through co-selection or cross-adaptation, although causal pathways require confirmation using genomic and functional approaches (Liu et al., 2017). Xinjiang is geographically expansive, and its healthcare delivery patterns and antimicrobial and disinfectant-use settings may differ from those of other regions. Nevertheless, the regional antimicrobial resistance profile, disinfectant susceptibility, and resistance-gene distribution of CRPA have not been systematically characterized (Barth et al., 2025).

We therefore evaluated antimicrobial susceptibility, MICs and MBCs for four commonly used disinfectants, and resistance-associated genes among CRPA isolates collected from two tertiary hospitals in Xinjiang. Phenotypes were compared between XDR and non-XDR isolates and between qacEΔ1-sul1-positive and -negative isolates. This study was designed to strengthen regional surveillance evidence and support antimicrobial stewardship and healthcare-associated infection prevention.

## 2 Materials and methods

### 2.1 Bacterial isolates

A total of 80 nonduplicate CRPA isolates were consecutively collected from clinical specimens at two tertiary hospitals in Urumqi between January 2025 and March 2026. The two participating institutions are the Fifth Affiliated Hospital of Xinjiang Medical University, a general tertiary hospital with 1,200 authorised beds, and the Fourth Affiliated Hospital of Xinjiang Medical University, a tertiary traditional Chinese medicine hospital with 2,400 authorised beds, both located in Urumqi. Only the first CRPA isolate from each patient was retained, applied across the entire study period rather than within calendar sub-periods, so that each of the 80 isolates derives from a distinct patient. All isolates meeting these criteria were enrolled consecutively without selective exclusion. Species identification was performed using the VITEK 2 automated microbial identification system (bioMérieux, France). P. aeruginosa ATCC 27853 and Escherichia coli ATCC 25922 served as quality control strains for both identification and susceptibility testing. ATCC 27853 was processed alongside clinical isolates in each VITEK 2 identification batch to verify system performance, and correct identification to species level was confirmed in every run. The VITEK 2 system undergoes routine internal quality assurance in accordance with the participating hospitals’ laboratory accreditation requirements, including daily system checks and periodic proficiency testing. Carbapenemase production was assessed by the modified carbapenem inactivation method according to CLSI M100 (2021) (Humphries et al., 2021). A meropenem disk incubated with a bacterial suspension for 4 h was transferred to a lawn of E. coli ATCC 25922; a zone diameter of 6–15 mm, or the presence of colonies within a 16–18 mm zone, was recorded as positive, and a zone of at least 19 mm as negative. Klebsiella pneumoniae ATCC BAA-1705 and ATCC BAA-1706 were included in each run as positive and negative controls respectively. This study was approved by the Ethics Committee of the Fifth Affiliated Hospital of Xinjiang Medical University (approval no. XYDWFYLsk-2025-4; approval date 6 January 2025).

### 2.2 Antimicrobial susceptibility testing

Antimicrobial susceptibility was primarily determined using the VITEK 2 AST-GN card (bioMérieux, France), which tests the following agents at the indicated concentration ranges: ticarcillin/clavulanate (8/2–128/2 μg/mL), piperacillin/tazobactam (TZP; 4/4–128/4 μg/mL), ceftazidime (CAZ; 1–64 μg/mL), cefepime (FEP; 1–64 μg/mL), cefoperazone/sulbactam (8/4–64/4 μg/mL), imipenem (IPM; 1–16 μg/mL), meropenem (MEM; 0.25–16 μg/mL), amikacin (AMK; 2–64 μg/mL), tobramycin (TOB; 1–16 μg/mL), levofloxacin (LVX; 0.25–8 μg/mL), and ciprofloxacin (0.25–4 μg/mL). Aztreonam (ATM) was tested by the Kirby–Bauer disk diffusion method using a 30 μg disk (Oxoid, UK), which was also used to verify isolates with discordant VITEK 2 results. P. aeruginosa ATCC 27853 served as the quality control strain for each testing batch. Results were interpreted according to CLSI M100-S31 (2021) (Humphries et al., 2021), the edition in force in both participating laboratories throughout the collection period, with testing performed prospectively as isolates were recovered. The interpretive criteria for all agents tested against P. aeruginosa were subsequently re-examined against CLSI M100-Ed35 (2025) (Lewis, 2025); no applicable breakpoint changed in a manner that would alter any susceptibility interpretation or any MDR or XDR assignment. Colistin and other polymyxins were not tested and did not contribute to the MDR or XDR classification. Throughout this manuscript, “resistant” denotes category R only, and “nonsusceptible” denotes intermediate and resistant combined. Multidrug-resistant (MDR) and XDR definitions followed the international expert consensus of Magiorakos et al. (2012): MDR was defined as nonsusceptibility to at least one agent in three or more antimicrobial categories, and XDR as nonsusceptibility to all categories except one or two. All tables report R, I and S categories separately. The manual broth microdilution method described in Sections 2.4 and 2.5 was applied exclusively to disinfectant MIC and MBC determination and was not used for antimicrobial susceptibility testing.

### 2.3 Disinfectants

Four commonly used clinical disinfectants were employed: benzalkonium bromide (Shanghai Macklin Biochemical Co., Ltd., Shanghai, China) as a low-level disinfectant; ethanol and povidone-iodine (both from Beijing Xidebao Disinfection Products Co., Ltd., Beijing, China) as intermediate-level disinfectants; and a chlorine-containing disinfectant (Shandong Jiajie Water Purification Technology Co., Ltd., Shandong, China) as a high-level disinfectant. All working solutions were freshly prepared immediately prior to each experiment.

Stock solutions were normalised to defined active-ingredient concentrations before serial dilution. Benzalkonium bromide was prepared as a 512 mg/L active-ingredient stock from the 0.1% (equivalent to 1000 mg/L) product, verified against the manufacturer’s certificate of analysis. Ethanol was used at 75% (v/v) as the highest test level, equivalent to approximately 5.9 × 10^5^ mg/L given a density of 0.789 g/mL. Povidone-iodine was prepared at 1024 mg/L available iodine from the 1% product. The chlorine-containing disinfectant was prepared at 1024 mg/L available chlorine, verified by iodometric titration. The conversion used throughout was C (mg/L) = 10 000 × w/w %. Because ethanol is prepared and used volumetrically, its endpoints are reported as volume fractions rather than mass concentrations.

### 2.4 Determination of disinfectant MICs

This assay was adapted from antimicrobial broth microdilution and is not a standardised biocide efficacy test. It was used solely to compare relative *in vitro* tolerance between isolate groups examined under identical conditions, and the resulting MIC and MBC values are not equivalent to, and cannot be substituted for, the endpoints of suspension or carrier tests such as EN 13727, EN 13697, or AOAC methods. Stock solutions were subjected to twofold serial dilution in cation-adjusted Mueller–Hinton broth in 96-well microplates to generate eight successive concentration gradients. The complete series were: benzalkonium bromide 512, 256, 128, 64, 32, 16, 8, and 4 mg/L; ethanol 75, 37.5, 18.75, 9.375, 4.69, 2.34, 1.17, and 0.59% (v/v); povidone-iodine 1024, 512, 256, 128, 64, 32, 16, and 8 mg/L available iodine; and the chlorine-containing disinfectant 1024, 512, 256, 128, 64, 32, 16, and 8 mg/L available chlorine. Test strains were suspended in sterile normal saline and adjusted to 0.5 McFarland standard, approximately 1 × 10^8^ CFU/mL. An equal volume of bacterial suspension was added to each well, yielding a final inoculum of approximately 5 × 10^5^ CFU/mL. After gentle mixing, microplates were sealed and incubated at 37 °C for 18–24 h under aerobic static conditions. The MIC was defined as the lowest concentration exhibiting no visible turbidity. Sterile distilled water without bacteria served as the negative control, and bacterial suspension in disinfectant-free broth as the positive growth control. P. aeruginosa ATCC 27853 was tested alongside each batch. All experiments were independently repeated three times on separate days; results were accepted when the three replicates agreed within one twofold dilution step, and otherwise additional replicates were performed and the modal value recorded.

### 2.5 Determination of disinfectant MBCs

The MBC was determined from the completed MIC microdilution plates. A volume of 100 μL was aspirated from every well exhibiting no visible growth and transferred into neutralisation broth containing 3% lecithin and 5% polysorbate 80 in phosphate-buffered saline, formulated to inactivate residual quaternary ammonium compounds, ethanol, povidone-iodine, and chlorine-containing disinfectant (Espigares et al., 2003). The mixture was held at room temperature for 10 min, after which 100 μL of neutralised suspension was spread onto Columbia blood agar and incubated at 37 °C for 18–24 h. Prior to formal testing the neutralising medium was validated by two controls. First, a neutraliser efficacy control, in which the neutralising medium was combined with each of the four disinfectants at the highest tested concentration, held at room temperature for 10 min, and then inoculated with a standardised suspension of the test organism; recovery within 0.5 log_10_ of the growth control confirmed effective quenching of residual activity for all four agents, including the chlorine-containing disinfectant and povidone-iodine. Second, a neutraliser toxicity control, in which the neutralising medium alone was inoculated under identical conditions; recovery within 0.5 log_10_ of the growth control confirmed the absence of bacteriostatic or bactericidal activity attributable to the neutraliser itself (Espigares et al., 2003).Because 100 μL of neutralised suspension was plated, the limit of detection of this procedure was 10 CFU/mL. The endpoint recorded is therefore defined as the lowest concentration producing no detectable growth on the plated volume, corresponding to a reduction of at least approximately 4.7 log_10_ from the starting inoculum. It is designated the MBC for consistency with the prior literature but was not established by quantitative log-reduction counting, and initial viable counts and recovery efficiency were not determined for individual isolates.

### 2.6 Genomic DNA extraction and PCR amplification

Genomic DNA was extracted using the boiling lysis method, which has been validated for reliable extraction of templates suitable for PCR-based resistance gene detection in Gram-negative bacteria including P. aeruginosa (Edelstein et al., 2013; Loberiza et al., 2025). Single colonies were suspended in 500 μL sterile double-distilled water, incubated in a 100 °C water bath for 10–15 min, and centrifuged at 12,000 r/min for 5 min; the supernatant containing crude genomic DNA was collected and stored at −20 °C as the PCR template. PCR was performed to screen carbapenem resistance-associated genes (blaKPC, blaNDM, oprD, blaOXA-10, blaOXA-48, blaVIM, blaIMP) and disinfectant resistance genes (qacEΔ1-sul1, aceI, emrE, merA, tehA, oqxA). Primer sequences, product lengths, and annealing temperatures were taken from published literature (Li and Zhang, 2024; Liang, 2025) and are detailed in **Tables 1**, **2**. All primer sequences were re-checked against the primary sources cited and verified in silico using NCBI Primer-BLAST against reference sequences for each target, confirming agreement between predicted and reported amplicon lengths and the absence of predicted non-target amplification within the P. aeruginosa genome or across the Enterobacterales. PCR was carried out in a 25 μL system containing 12.5 μL 2× Taq PCR Master Mix, 1 μL each of forward and reverse primers (10 μmol/L), 2 μL DNA template, and 8.5 μL nuclease-free water. Cycling conditions were 94 °C for 5 min; 35 cycles of 94 °C for 30 s, gene-specific annealing for 30 s, and 72 °C for 40 s; and a final extension at 72 °C for 10 min. Products (5 μL) were electrophoresed on 1% GoldView agarose gel at 120 V for 30 min and documented by gel imaging. Each run included a target-specific positive control, a no-template negative control, and P. aeruginosa ATCC 27853 as a negative reference strain. Amplicon identity was confirmed by product size relative to the positive control; PCR products from clinical isolates were not sequenced. Failure to amplify the expected oprD target is compatible with primer-binding site variation, insertional disruption, structural rearrangement, suboptimal template quality, or technical failure, and does not distinguish among these possibilities. It is reported throughout as an amplification result and not as evidence of gene deletion, protein loss, or reduced expression.

**Table 1:** Primers used to detect disinfectant resistance genes.

Gene	Forward primer (5′–3′)	Reverse primer (5′–3′)	Product (bp)	Annealing temperature (°C)
aceI	CGGGCATTAATCTCGATCTGTACA	GCAGCTCACAGACCACAACAATAGT	966	58
emrE	ATGACCAACTATCTCTACCTCGC	TCAGTGCACCGAAGCGCGGA	333	55
merA	GACCACGCCGAGTTGCGTCTA	GCAGCAAGAAAGCTGCTTCA	462	62
qacEΔ1-sul1	TAGCGAGGGCTTTACTAAGC	ATTCAGAATGCCGAACACCG	300	57
tehA	ATGCAGAGCGATAAAGTGCTC	TGGACTGTATCTGCCGACAG	436	55
oqxA	GCGCTCGGCGCGATGATGCTC	CTGCTCCCACTCTTCACGGGA	402	55

All primer sequences were re-verified against the primary sources cited and confirmed in silico by NCBI Primer-BLAST against the corresponding reference sequences; predicted amplicon lengths correspond to those listed.

**Table 2:** Primers used to detect antimicrobial resistance-associated genes.

Gene	Forward primer (5′–3′)	Reverse primer (5′–3′)	Product (bp)	Annealing temperature (°C)
blaKPC	GTCTAGTCTGCTGTCTTG	CTTGTCATCCTTGTTAGGCG	920	56
blaNDM	CTCTGTCACATCGAAATCGC	CACCTCATGTTGAATTCGCC	984	56
oprD	GCGCATCTCCAAGACCATG	GCCACGCGATTTGACGGAG	193	54
blaOXA-10	TCTTTCGAGTACGGCATTAGC	CCAATGATGCCCTCACCTTTCC	760	56
blaOXA-48	CATCAAGTTCAACCCAACCG	GCGTGGTTAAGGATGAACAC	942	56
blaVIM	ATTCCGGTCGGAGAGGTCG	GAGCAAGTCTAGACCGCCCG	633	56
blaIMP	CGGCCTCAGGAGAGTCTTT	AACCAGTTTGCTTTACCAT	587	56

All primer sequences were re-verified against the primary sources cited and confirmed in silico by NCBI Primer-BLAST against the corresponding reference sequences; predicted amplicon lengths correspond to those listed.

### 2.7 Statistical analysis

Statistical analyses were performed using SPSS version 26.0 and Python 3.11 (SciPy). Non-normally distributed continuous variables are presented as medians with interquartile ranges and were compared using the Mann–Whitney U test; for benzalkonium bromide the Hodges–Lehmann median difference and an effect-size estimate are also reported. Categorical variables are presented as counts and percentages and were compared using the χ² test where all expected cell frequencies were at least five, and Fisher’s exact test otherwise; the test used is indicated for each comparison. Odds ratios with 95% confidence intervals are reported only where the comparison is not definitional. All tests were two-sided. Outcomes were organised into one primary family and three exploratory families. This hierarchy was specified during revision rather than before data analysis and is therefore *post hoc*. The primary family comprised the two comparisons of benzalkonium bromide MIC and MBC between XDR and non-XDR isolates, to which the Holm procedure was applied. The exploratory families comprised the nine comparisons of antimicrobial resistance by qacEΔ1-sul1 status, the eight comparisons of resistance-gene distribution by XDR status, and the six comparisons of the remaining three disinfectants; within each, the Benjamini–Hochberg false discovery rate procedure was applied and q values are reported alongside unadjusted P values. Holm-adjusted values are additionally reported for the qacEΔ1-sul1 family as a sensitivity analysis. A sensitivity analysis treating all eight disinfectant endpoints as a single family is also reported. No multivariable model of XDR status was fitted. Resistance to individual antimicrobial agents forms part of the XDR definition under the Magiorakos criteria, so regressing XDR status on those variables would be circular; in addition, with 24 XDR isolates, a model containing three covariates would provide approximately eight events per variable, below accepted minima for stable estimation. Comparisons of individual antimicrobial agents between XDR and non-XDR isolates are therefore reported descriptively, as absolute rates and rate differences, and are not interpreted as independent associations.

## 3 Results

### 3.1 Clinical characteristics of the CRPA isolates

During the 15-month study period, approximately 320 P. aeruginosa clinical isolates were recovered from the two participating hospitals, of which 80 (25.0%) met the CRPA criteria and were included. The 80 isolates derived from 80 individual patients. Median patient age was 65 years (interquartile range 52–76), with 51 males (63.75%) and 29 females (36.25%). Most patients (72.50%) had at least one underlying comorbidity, including chronic pulmonary disease, cardiovascular disease, diabetes mellitus, or malignancy. Median length of hospital stay prior to CRPA isolation was 14 days (interquartile range 8–25).Sputum was the most common specimen type (52.50%, 42/80), followed by bronchoalveolar lavage fluid (20.00%, 16/80) and urine (17.50%, 14/80). The remaining eight isolates were recovered from whole blood, throat swabs, pus, bronchoscopic brush specimens, and vaginal secretions, each accounting for no more than 5% of the total. Forty-eight isolates (60.0%) were recovered from the Fifth Affiliated Hospital and 32 (40.0%) from the Fourth Affiliated Hospital. Comparison between centres showed no material differences in the XDR proportion (29.2% versus 31.3%), qacEΔ1-sul1 carriage (37.5% versus 34.4%), failure to amplify oprD (70.8% versus 65.6%), or benzalkonium bromide MIC and MBC distributions (all P > 0.05). The absence of a detectable between-hospital difference does not exclude clonal clustering at ward level. The isolates originated from 17 clinical departments (**Figure 1**). The intensive care unit contributed the largest number (n = 21, 26.25%), followed by respiratory and critical care medicine (n = 15, 18.75%), infectious diseases (n = 7, 8.75%), and cardiology (n = 5, 6.25%). Orthopaedics, neurology, neurosurgery, urology, rehabilitation medicine, and the emergency department each contributed three isolates (3.75% each). Haematology and oncology, nephrology, general practice, geriatrics, cardiac surgery, endocrinology, and hepatobiliary surgery each contributed two isolates (2.50% each).

**Figure 1:**
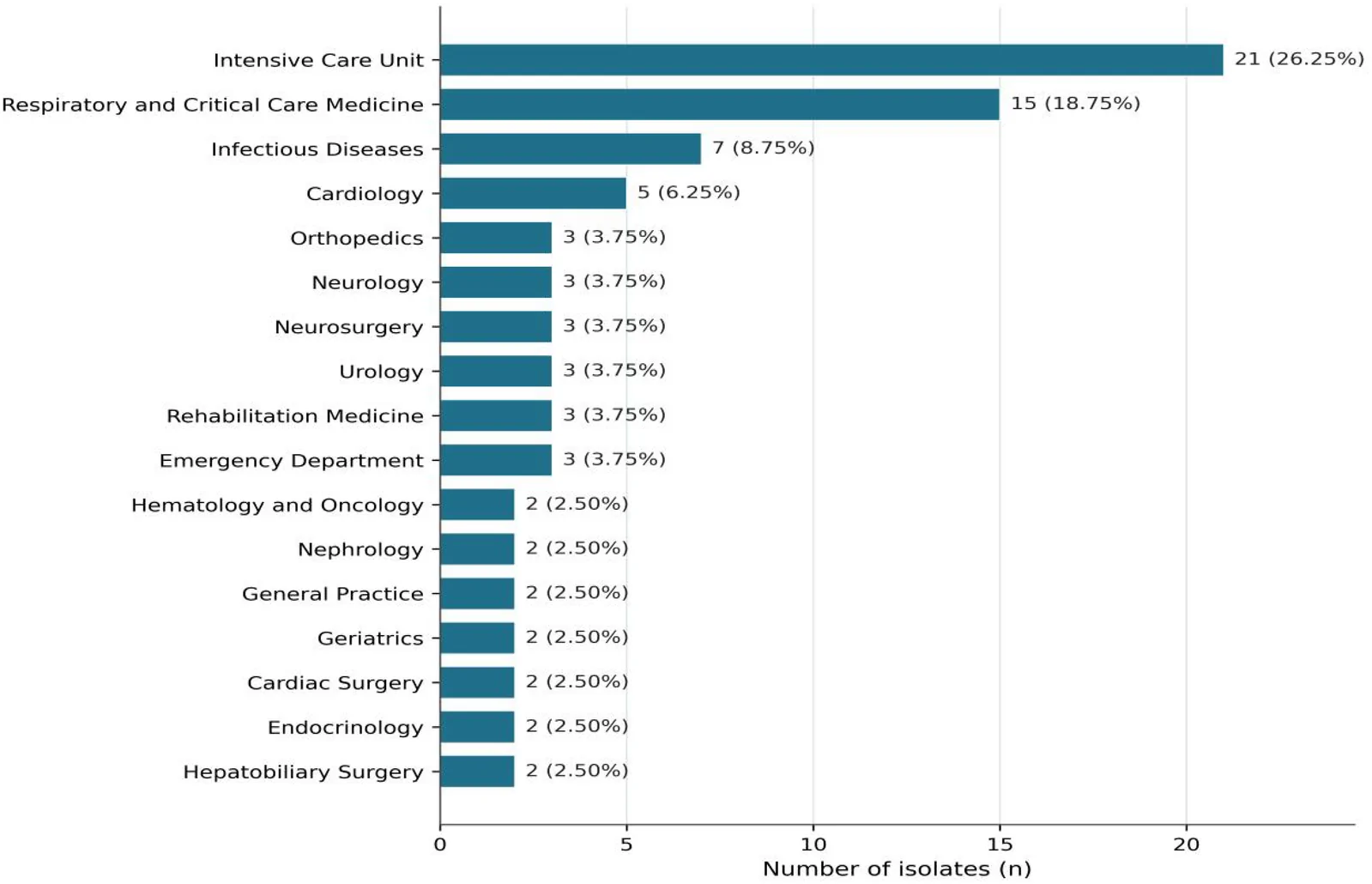
Distribution of CRPA isolates by clinical department. Eighty CRPA isolates were recovered from 17 clinical departments. Bars show the number of isolates and the percentage of all isolates contributed by each department. CRPA, carbapenem-resistant Pseudomonas aeruginosa; n = 80.

### 3.2 Antimicrobial susceptibility

Among the 80 CRPA isolates, 24 were XDR and 56 were non-XDR. Resistance rates to imipenem and meropenem were 83.7% and 68.7% respectively, and no isolate was susceptible to either agent. Resistance rates to ceftazidime, cefepime, piperacillin/tazobactam, aztreonam, and levofloxacin were 70.8%, 66.7%, 75.0%, 70.8%, and 58.3% respectively among XDR isolates, compared with 5.4%, 1.8%, 10.7%, 23.2%, and 12.5% among non-XDR isolates. Resistance to amikacin and tobramycin was 33.3% for both agents in the XDR group, compared with 3.6% and 1.8% respectively in the non-XDR group. These differences are definitional rather than empirical, because nonsusceptibility to these agents contributes to the XDR classification, and they are therefore presented descriptively without measures of association or hypothesis tests. Detailed results are presented in **Table 3**.

**Table 3:** Antimicrobial susceptibility profiles of 80 CRPA isolates.

Antimicrobial	Overall R (%)	Overall I (%)	Overall S (%)	XDR R (%)	XDR I (%)	XDR S (%)	Non-XDR R (%)	Non-XDR I (%)	Non-XDR S (%)	Rate difference in R, % (95% CI)
AMK	12.50	5.00	82.50	33.30	16.70	50.00	3.60	0.00	96.40	29.7 (10.2 to 49.2)
LVX	26.30	20.00	53.70	58.30	37.50	4.20	12.50	12.50	75.00	45.8 (24.3 to 67.3)
TZP	30.00	11.30	58.70	75.00	20.80	4.20	10.70	7.10	82.20	64.3 (45.2 to 83.4)
ATM	37.50	22.50	40.00	70.80	8.40	20.80	23.20	28.60	48.20	47.6 (26.3 to 68.9)
CAZ	25.00	12.50	62.50	70.80	20.80	8.40	5.40	8.90	85.70	65.4 (46.3 to 84.5)
FEP	21.30	16.30	62.40	66.70	25.00	8.30	1.80	12.50	85.70	64.9 (45.7 to 84.1)
IPM	83.70	16.30	0.00	91.70	8.30	0.00	80.40	19.60	0.00	11.3 (−3.9 to 26.5)
MEM	68.70	31.30	0.00	91.70	8.30	0.00	58.90	41.10	0.00	32.8 (15.8 to 49.8)
TOB	6.30	11.30	82.40	33.30	4.20	62.50	1.80	1.80	96.40	31.5 (12.3 to 50.7)

Resistance to the agents listed contributes to the XDR classification under the Magiorakos criteria. Differences between XDR and non-XDR isolates are therefore definitional and are presented for description only; no measures of association or hypothesis tests are reported for these comparisons. Rate differences refer to category R and are given with Wald 95% confidence intervals. R, resistant; I, intermediate; S, susceptible; CI, confidence interval; CRPA, carbapenem-resistant Pseudomonas aeruginosa; XDR, extensively drug-resistant; AMK, amikacin; LVX, levofloxacin; TZP, piperacillin/tazobactam; ATM, aztreonam; CAZ, ceftazidime; FEP, cefepime; IPM, imipenem; MEM, meropenem; TOB, tobramycin.

### 3.3 Detection of antimicrobial and disinfectant resistance genes

The modified carbapenem inactivation method was positive for 23 of 80 isolates (28.75%). All 19 isolates carrying at least one of the four carbapenemase genes detected were mCIM-positive, and a further four isolates were mCIM-positive without any of the seven genes sought. Agreement between the phenotypic and genotypic results was 95.0% (76/80), with Cohen’s κ 0.871 (95% CI 0.748–0.994). Because the PCR panel covered seven targets only, it does not constitute a reference standard, and measures of diagnostic accuracy such as sensitivity and specificity are not reported; the four discordant isolates are compatible with carbapenemases encoded by genes outside the panel, although this was not investigated. The expected oprD amplicon failed to amplify in 55 of 80 isolates (68.75%). The carbapenemase genes blaKPC, blaNDM, blaVIM, and blaIMP were detected in 10.00% (8/80), 6.25% (5/80), 5.00% (4/80), and 2.50% (2/80) of isolates respectively, with no co-carriage observed. Neither blaOXA-10 nor blaOXA-48 was detected. Among disinfectant resistance genes, qacEΔ1-sul1 was the most frequently detected (36.25%, 29/80), followed by merA (16.25%, 13/80), tehA (7.50%, 6/80), and oqxA (2.50%, 2/80). Neither aceI nor emrE was detected.

qacEΔ1-sul1 was detected in 58.3% (14/24) of XDR isolates and 26.8% (15/56) of non-XDR isolates (OR 3.83, 95% CI 1.40–10.45; unadjusted Fisher’s exact P = 0.011). This association did not retain statistical significance after correction within the family of eight gene comparisons (Benjamini–Hochberg q = 0.088; Holm-adjusted P = 0.088). No other gene differed between groups. Detailed results are shown in **Table 4** and **Figure 2**.

**Table 4:** Distribution of resistance-associated genes in XDR and non-XDR isolates.

Gene category	Gene	XDR-positive (n=24), n (%)	Non-XDR-positive (n=56), n (%)	OR (95% CI)	Unadjusted P	q value (BH-FDR)
Antimicrobial resistance-associated	blaKPC	4 (16.70)	4 (7.10)	2.60 (0.59–11.41)	0.232	0.723
Antimicrobial resistance-associated	blaNDM	2 (8.30)	3 (5.40)	1.61 (0.25–10.29)	0.633	0.723
Antimicrobial resistance-associated	oprD amplification failure	18 (75.00)	37 (66.10)	1.54 (0.51–4.67)	0.599	0.723
Antimicrobial resistance-associated	blaVIM	2 (8.30)	2 (3.60)	2.45 (0.33–18.54)	0.579	0.723
Disinfectant resistance	merA	5 (20.80)	8 (14.30)	1.58 (0.46–5.44)	0.516	0.723
Disinfectant resistance	qacEΔ1-sul1	14 (58.30)	15 (26.80)	3.83 (1.40–10.45)	0.011	0.088
Disinfectant resistance	tehA	2 (8.30)	4 (7.10)	1.18 (0.20–6.93)	1.000	1.000
Disinfectant resistance	oqxA	1 (4.20)	1 (1.80)	2.39 (0.14–39.89)	0.513	0.723

All P values were calculated using two-sided Fisher’s exact test. q values were calculated by the Benjamini–Hochberg false discovery rate procedure across the eight comparisons in this exploratory family; no association retained significance after correction (all q > 0.05). Holm-adjusted P for qacEΔ1-sul1 was 0.088. OR, odds ratio; CI, confidence interval; BH-FDR, Benjamini–Hochberg false discovery rate. “oprD amplification failure” denotes failure to amplify the expected PCR target and does not imply gene deletion or loss of protein expression.

**Figure 2:**
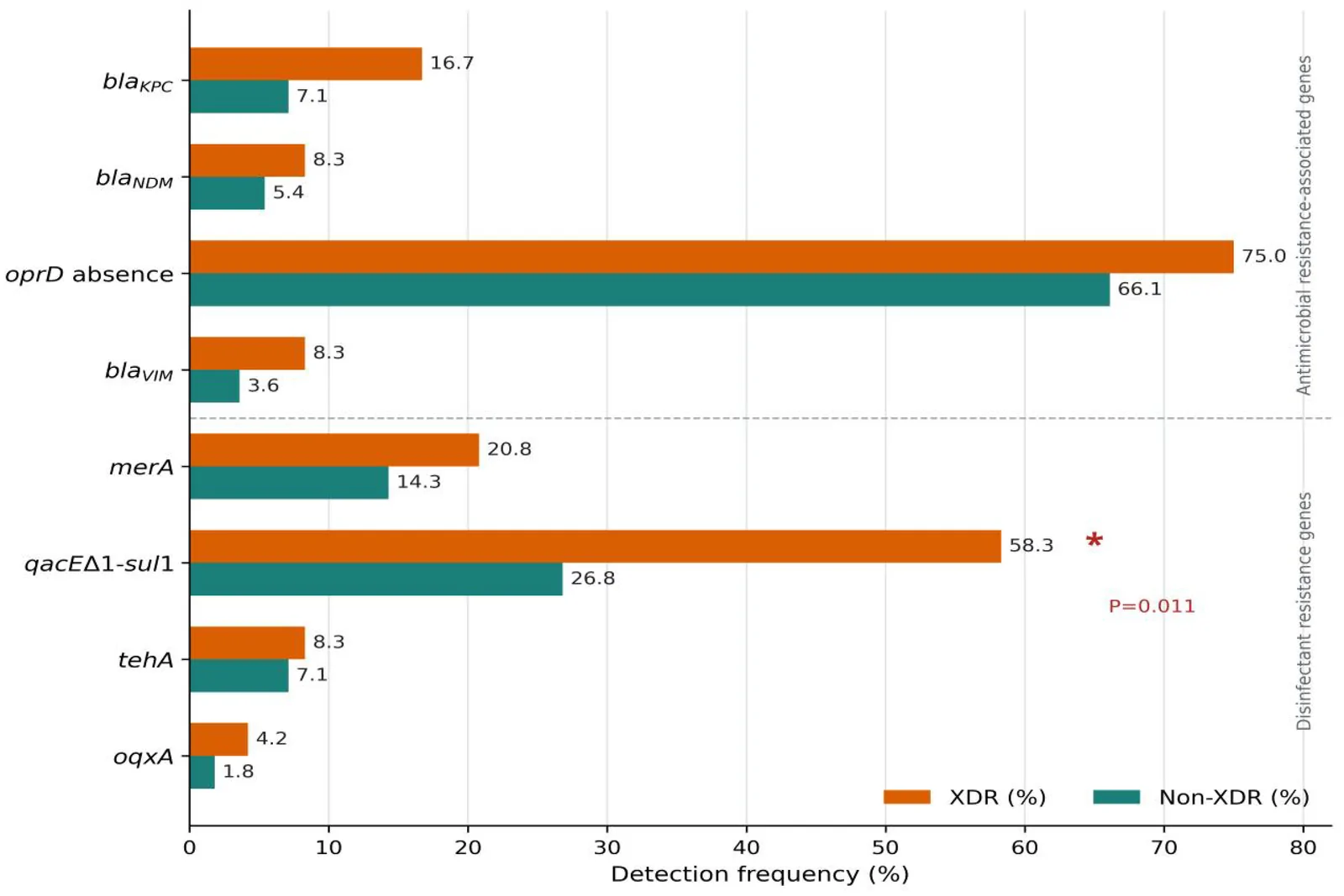
Detection frequencies of antimicrobial resistance-associated genes and disinfectant resistance genes among XDR and non-XDR CRPA isolates. The XDR group comprised 24 isolates and the non-XDR group 56 isolates. Comparisons used two-sided Fisher’s exact test with Benjamini–Hochberg correction across the eight comparisons shown. For qacEΔ1-sul1, OR 3.83 (95% CI 1.40–10.45), unadjusted *P = 0.011, q = 0.088. CRPA,* carbapenem-resistant *Pseudomonas aeruginosa; XDR,* extensively drug-resistant.

### 3.4 MICs and MBCs of the four disinfectants

Ethanol, povidone-iodine, and the chlorine-containing disinfectant showed no difference in MIC or MBC between XDR and non-XDR isolates (all unadjusted P > 0.05; all q > 0.05 within the six-test exploratory family). For benzalkonium bromide, which constituted the primary outcome family, the MIC range was 32–256 mg/L in the XDR group and 16–256 mg/L in the non-XDR group, with the difference remaining significant after Holm correction within the two-test primary family (unadjusted P < 0.001; Holm-adjusted P < 0.001). The MBC ranges were 64–512 and 64–256 mg/L respectively (unadjusted P = 0.036; Holm-adjusted P = 0.036 within the primary family). In a prespecified sensitivity analysis treating all eight disinfectant endpoints as a single family, the benzalkonium bromide MIC difference remained significant (q = 0.001) whereas the MBC difference did not (q = 0.144). The MBC finding is therefore reported as provisional. See **Table 5**.

**Table 5:** MICs and MBCs of four disinfectants against CRPA isolates.

Disinfectant	CRPA group	MIC range	MIC50	MIC90	Unadjusted P (MIC)	Adjusted P or q (MIC)	MBC range	MBC50	MBC90	Unadjusted P (MBC)	Adjusted P or q (MBC)
Ethanol, % (v/v)	XDR (n=24)	75–75	75	75	0.100	q = 0.220	75–75	75	75	0.257	q = 0.336
Ethanol, % (v/v)	Non-XDR (n=56)	37.5–75	75	75			37.5–75	75	75		
Chlorine-containing disinfectant, mg/L available chlorine	XDR (n=24)	128–1024	512	1024	0.956	q = 0.956	256–1024	1024	1024	0.294	q = 0.336
Chlorine-containing disinfectant, mg/L available chlorine	Non-XDR (n=56)	128–1024	512	1024			256–1024	1024	1024		
Povidone-iodine, mg/L available iodine	XDR (n=24)	128–1024	512	1024	0.283	q = 0.336	256–1024	512	1024	0.110	q = 0.220
Povidone-iodine, mg/L available iodine	Non-XDR (n=56)	128–1024	512	1024			256–1024	512	1024		
Benzalkonium bromide, mg/L	XDR (n=24)	32–256	64	128	<0.001	Holm P < 0.001	64–512	128	512	0.036	Holm P = 0.036
Benzalkonium bromide, mg/L	Non-XDR (n=56)	16–256	64	128			64–256	128	256		

Groups were compared using the Mann–Whitney U test. Benzalkonium bromide MIC and MBC constituted the prespecified primary outcome family of two tests, to which the Holm procedure was applied; the remaining six comparisons constituted an exploratory family, to which the Benjamini–Hochberg procedure was applied. In a sensitivity analysis treating all eight endpoints as a single family, the benzalkonium bromide MIC difference remained significant (q = 0.001) whereas the MBC difference did not (q = 0.144). Ethanol endpoints are reported as volume fractions because the product is prepared and used volumetrically. The chlorine-containing disinfectant concentration label has been corrected from 2000 mg/L to 1024 mg/L available chlorine, the concentration actually tested. For benzalkonium bromide MIC, the Hodges–Lehmann median difference was 32.0 mg/L (95% CI 0.0–48.0), with a moderate-to-large effect size (r = 0.424). MIC, minimum inhibitory concentration; MBC, minimum bactericidal concentration.

### 3.5 Antimicrobial resistance and benzalkonium bromide endpoints according to qacEΔ1-sul1 status

In unadjusted analysis, resistance to aztreonam and meropenem was nominally more frequent among qacEΔ1-sul1-positive than among qacEΔ1-sul1-negative isolates (55.2% versus 27.5%, unadjusted P = 0.016; and 86.2% versus 58.8%, unadjusted P = 0.011). Neither association retained significance after correction within this nine-test exploratory family (Benjamini–Hochberg q = 0.072 for both; Holm-adjusted P = 0.111 and 0.100 respectively). No other agent differed between groups. Detailed results are presented in **Table 6**. Direct comparison of benzalkonium bromide endpoints by qacEΔ1-sul1 status showed no difference: median MIC was 64 mg/L in both groups (P = 0.097) and median MBC was 128 mg/L in both groups (P = 0.697). The null result was consistent when the comparison was stratified by hospital. Carriage of qacEΔ1-sul1 was therefore not associated with reduced benzalkonium bromide susceptibility in this collection.

**Table 6:** Antimicrobial resistance rates according to *qacEΔ1-sul1* status.

Antimicrobial	qacEΔ1-sul1-positive (n=29), resistant n (%)	qacEΔ1-sul1-negative (n=51), resistant n (%)	Test statistic	Unadjusted P	q value (BH-FDR)	Holm-adjusted P
Cefepime (FEP)	7 (24.10)	10 (19.60)	χ² = 0.238	0.626	0.805	1.000
Ceftazidime (CAZ)	10 (34.50)	10 (19.60)	χ² = 2.035	0.154	0.346	0.838
Amikacin (AMK)	6 (20.70)	4 (7.80)	Fisher	0.101	0.303	0.664
Aztreonam (ATM)	16 (55.20)	14 (27.50)	χ² = 5.782	0.016	0.072	0.111
Piperacillin/tazobactam (TZP)	11 (37.90)	13 (25.50)	χ² = 1.319	0.251	0.450	1.000
Tobramycin (TOB)	4 (13.80)	5 (9.80)	Fisher	0.718	0.808	1.000
Levofloxacin (LVX)	8 (27.60)	13 (25.50)	χ² = 0.042	0.837	0.837	1.000
Meropenem (MEM)	25 (86.20)	30 (58.80)	χ² = 6.416	0.011	0.072	0.100
Imipenem (IPM)	26 (89.70)	41 (80.40)	χ² = 1.075	0.300	0.450	1.000

Fisher’s exact test was used for amikacin and tobramycin because expected cell frequencies fell below five; the χ² test was used otherwise. q values were calculated by the Benjamini–Hochberg false discovery rate procedure across all nine comparisons in this exploratory family. Holm-adjusted P values are reported as a sensitivity analysis. After correction by either procedure, no association retained statistical significance. BH-FDR, Benjamini–Hochberg false discovery rate.

## 4 Discussion

### 4.1 Interpretation of antimicrobial resistance phenotypes

Among 80 CRPA isolates, 24 (30.00%) met the XDR definition. Imipenem and meropenem resistance rates were 83.7% and 68.7% respectively, with no susceptible isolates, indicating uniform nonsusceptibility to carbapenems in CRPA from these two hospitals. Resistance rates to β-lactams, β-lactam/β-lactamase-inhibitor combinations, fluoroquinolones, and aminoglycosides were substantially higher among XDR than non-XDR isolates, but this contrast follows from the XDR definition itself and should not be read as an empirical finding. The practical implication is that carbapenems should not be used as empirical monotherapy for local CRPA infections without susceptibility evidence, and that other agents should likewise be selected on individualised susceptibility results rather than on aggregate susceptibility rates (Loredo-Puerta et al., 2026). The XDR proportion observed here is comparable to reports from other regional hospitals (Huang et al., 2024), and the resistance pattern of XDR isolates is consistent with the multimechanism basis of CRPA described domestically and internationally (Strateva et al., 2024). International genomic surveillance indicates that extensive drug resistance in CRPA is typically not determined by a single gene but results from the superposition of porin abnormalities, efflux pump overexpression, AmpC dysregulation, and acquired carbapenemases (Guo et al., 2019). Stratifying CRPA into XDR and non-XDR therefore helps describe local resistance burden, but its clinical significance must be interpreted alongside infection site, drug exposure, pharmacokinetics and pharmacodynamics, and patient condition (Li et al., 2025).

### 4.2 Interpretation of disinfectant susceptibility

Among the four disinfectants, only benzalkonium bromide showed a difference between XDR and non-XDR isolates that survived correction for multiple comparisons, and only for the MIC endpoint; the MBC difference did not survive when all eight disinfectant endpoints were treated as a single family and is reported as provisional. The MIC finding is consistent with observations of reduced quaternary ammonium susceptibility in some multidrug-resistant Gram-negative bacteria (Khalili et al., 2022; Huang et al., 2026), and suggests that the XDR phenotype may co-occur with modestly reduced *in vitro* benzalkonium bromide susceptibility. The data demonstrate a difference in distribution between groups and nothing further; they cannot support any conclusion about performance of the product in clinical use (Behboudipour et al., 2025). Ethanol, povidone-iodine, and the chlorine-containing disinfectant showed no difference between groups. Their broad, non-specific bactericidal mechanisms may render endpoint results less sensitive to individual resistance determinants, but we did not measure membrane damage, oxidative stress, or efflux activity, and this explanation remains a hypothesis drawn from prior work rather than a finding of the present study (Abdelraheem et al., 2024; Gondal et al., 2024). *In vitro* MIC and MBC values in mg/L cannot be equated with labelled product concentrations, and they cannot be used to alter manufacturer-specified use concentrations or contact times. Infection-control practice should follow current standards and manufacturer instructions, with actual effectiveness assessed through standardised suspension or carrier tests, on-site microbiological monitoring, and process quality control (Liu et al., 2023).

### 4.3 Interpretation of antimicrobial and disinfectant resistance genes

Failure to amplify the expected oprD target was the most frequent molecular observation, occurring in 68.75% of isolates, whereas detection rates for blaKPC, blaNDM, blaVIM, and blaIMP were all low. It is tempting to read this distribution as evidence that OprD abnormality underlies carbapenem nonsusceptibility in this collection, and we deliberately refrain from doing so. Endpoint PCR non-amplification is compatible with primer-binding site variation, insertional disruption, structural rearrangement, template quality, or technical failure, and cannot be equated with gene deletion, protein absence, or reduced expression. Establishing any relationship between oprD status and carbapenem MIC would require sequencing, transcriptional quantification, and protein-level assessment, none of which was performed here. The mCIM results add a useful phenotypic perspective: 23 isolates showed carbapenemase activity, four of them without any of the seven genes sought, indicating that our panel does not capture the full carbapenemase repertoire of this collection. Detection of qacEΔ1-sul1 by PCR establishes the presence of the genetic element and nothing about its transcription, translation, or phenotypic effect. qacEΔ1-sul1 had the highest detection rate among disinfectant resistance genes (36.25%) and was more frequent among XDR isolates in unadjusted analysis, although this association did not survive correction for multiple comparisons (Sonbol et al., 2019; Kayet and Basak, 2026). Because qacEΔ1-sul1 is commonly located in the 3′ conserved segment of class 1 integrons, its detection may mark a genetic background carrying multiple resistance determinants rather than an independent contributor to the XDR phenotype; we did not characterise integron structure and therefore cannot test this (Ma et al., 2024). Low detection rates of merA, tehA, and oqxA, and the absence of aceI and emrE, indicate that the disinfectant resistance gene profile of this collection is dominated by qacEΔ1-sul1, though efflux and regulatory genes not screened here may also contribute (Sheikh et al., 2019; Zhang et al., 2023; Cai et al., 2024; Du and Liu, 2025; Xiao et al., 2025).

### 4.4 Relationship between antimicrobial resistance and disinfectant tolerance

The relationship we had originally proposed between qacEΔ1-sul1 carriage and reduced benzalkonium bromide susceptibility is not supported by direct testing. Median MIC and MBC values were identical in qacEΔ1-sul1-positive and -negative isolates, and the null result held when stratified by hospital. The nominal associations between qacEΔ1-sul1 carriage and aztreonam or meropenem resistance did not survive correction for multiple comparisons by either the Benjamini–Hochberg or the Holm procedure. Two observations therefore remain: XDR isolates had higher benzalkonium bromide MICs, and qacEΔ1-sul1 was more common among XDR isolates in unadjusted analysis. These cannot be joined into a mechanistic account. The elevated benzalkonium bromide MICs in the XDR group are thus unexplained by the determinants we measured. Plausible contributors include overexpression of intrinsic RND-family efflux systems such as MexAB-OprM, or altered membrane permeability, both of which are known to affect quaternary ammonium susceptibility in P. aeruginosa; neither was measured here and both remain hypotheses (Ma et al., 2024). Establishing whether qacEΔ1-sul1 or any other determinant contributes functionally would require transcriptional profiling, efflux activity assays, targeted gene inactivation, and whole-genome sequencing with molecular typing to control for clonal structure.

### 4.5 Clinical and infection-control implications

CRPA from these two hospitals showed considerable heterogeneity in resistance, with XDR isolates having more limited therapeutic options; this supports stratified surveillance and susceptibility-guided therapy rather than empirical carbapenem monotherapy. For infection control, *in vitro* disinfectant MIC and MBC values cannot justify altering labelled product concentrations or contact times. The reduced *in vitro* benzalkonium bromide susceptibility of XDR isolates is best interpreted as a signal favouring attention to preparation accuracy, adherence to labelled contact time, surface pre-cleaning, and periodic on-site verification of disinfection effectiveness; it is not evidence of clinical product failure.

### 4.6 Limitations

This study has several limitations. First, the modest sample (80 CRPA isolates from two Urumqi tertiary hospitals) limits extrapolation to Xinjiang or broader regions. Second, because all isolates were collected from two hospitals in the same city without molecular typing (e.g., MLST or WGS), we cannot exclude the possibility that the observed resistance patterns and gene-phenotype associations partially reflect the dissemination of one or more dominant clones rather than independent genetic acquisition events. Third, the study focused on select carbapenemases and oprD, without assessing intrinsic efflux pump expression (e.g., MexAB-OprM) or chromosomal AmpC derepression, which are major contributors to P. aeruginosa resistance. Fourth, due to the lack of detailed clinical follow-up, we could not strictly distinguish between infection and colonization, which may affect the assessment of true clinical burden. Fifth, we employed a broth microdilution method adapted from antimicrobial susceptibility testing to evaluate disinfectants. This approach uses a high bacterial inoculum (5 × 10^5^ CFU/mL) and does not incorporate quantitative log-reduction calculations or standardized biocide testing conditions (e.g., EN 13727 or carrier tests); therefore, the results reflect *in vitro* tolerance trends and cannot be directly extrapolated to clinical disinfection efficacy. The bactericidal endpoint was determined by the absence of colonies from a single plated volume rather than by quantitative viable counting; initial viable counts and recovery efficiency were not determined for individual isolates, and the endpoint therefore establishes a reduction below the limit of detection rather than a verified reduction of at least 5 log_10_.Future large-scale, multicentre genomic studies are needed to comprehensively map the resistome and clonal dynamics of CRPA in northwestern China.

## 5 Conclusions

This study provides a comprehensive characterisation of antimicrobial resistance phenotypes, disinfectant susceptibility, and resistance-associated gene carriage in 80 CRPA isolates from two tertiary hospitals in Urumqi, China. The principal findings are as follows. First, 30% of isolates were classified as XDR under the Magiorakos criteria, indicating a high burden of extensively drug-resistant P. aeruginosa in this clinical setting. Second, XDR isolates demonstrated significantly higher *in vitro* tolerance to benzalkonium bromide compared with non-XDR isolates (Holm-adjusted P < 0.001 for MIC), a robust phenotypic observation that warrants attention in infection control practice. Third, qacEΔ1-sul1 was detected in 36.25% of isolates and was more prevalent among XDR isolates in unadjusted analysis; however, direct comparison of benzalkonium bromide endpoints by qacEΔ1-sul1 status showed no significant difference, indicating that the functional contribution of this gene to the observed phenotype remains to be established by expression and functional studies. Fourth, failure to amplify the expected oprD target was observed in 68.75% of isolates, highlighting the potential importance of outer membrane porin-related mechanisms in carbapenem resistance in this region; the underlying basis of this observation requires further investigation by sequencing and protein-level methods. Fifth, carbapenemase gene carriage was detected in 23.75% of isolates, with blaKPC predominating, and mCIM results showed high concordance with PCR findings (95.0%). These findings provide valuable baseline epidemiological data for CRPA in Urumqi and may inform local infection control strategies and antimicrobial stewardship programmes. Future studies with larger sample sizes, molecular typing, functional gene expression analysis, and prospective design are needed to establish the mechanistic determinants of the observed resistance phenotypes and to enable generalisation beyond the two hospitals studied here.

## Data Availability

The datasets generated for this study contain clinical‑related isolate information that cannot be publicly released due to patient‑privacy‑related ethical restrictions. The minimal underlying datasets supporting the conclusions of this article are available from the corresponding author Ping Tian upon reasonable request via email at tianping19761976@163.com.
